# Supplementary material for: Effects of Trichoderma harzianum combined with Phanerochaete chrysosporium on lignin degradation and humification during chicken manure and rice husk composting
Source: Front Microbiol. 2025 Feb 28;16:1515931. doi: 10.3389/fmicb.2025.1515931 (PMC11906335; doi:10.3389/fmicb.2025.1515931)
Supplement: Supplementary file 1 [file Data_Sheet_1.zip › 6.Function/BFHS_BFHF/FunGuild/l1/BFHF_vs_BFHS_STAMP.pdf]

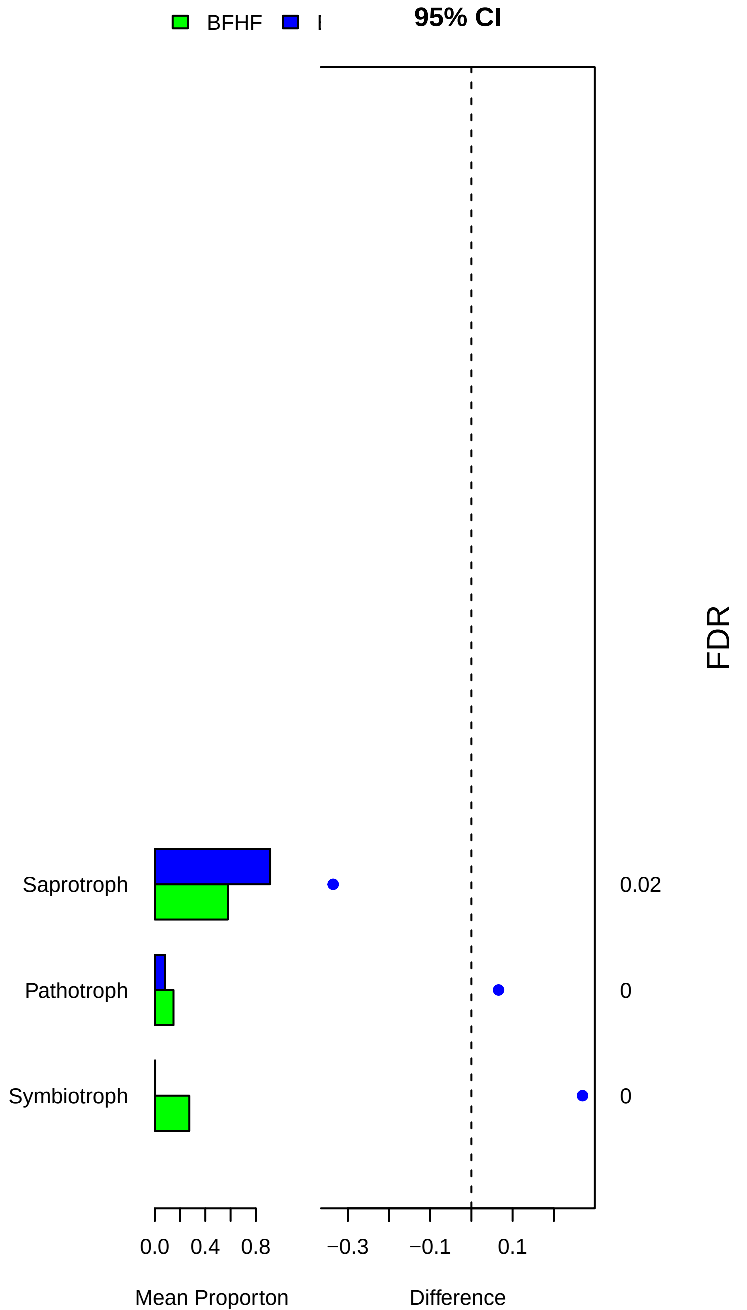

BFHF
95% CI
Saprotroph
Pathotroph
Symbiotroph
0.02
0
0
FDR
0.0
0.4
0.8
−0.3
−0.1
0.1
Mean Proporton
Difference
